# Supplementary material for: ERK Inhibition Increases RANKL-Induced Osteoclast Differentiation in RAW 264.7 Cells by Stimulating AMPK Activation and RANK Expression and Inhibiting Anti-Osteoclastogenic Factor Expression
Source: Int J Mol Sci. 2022 Nov 4;23(21):13512. doi: 10.3390/ijms232113512 (PMC9656104; doi:10.3390/ijms232113512)
Supplement: Supplementary file 1 [file ijms-23-13512-s001.zip › ijms-1880789-supplementary.pdf]

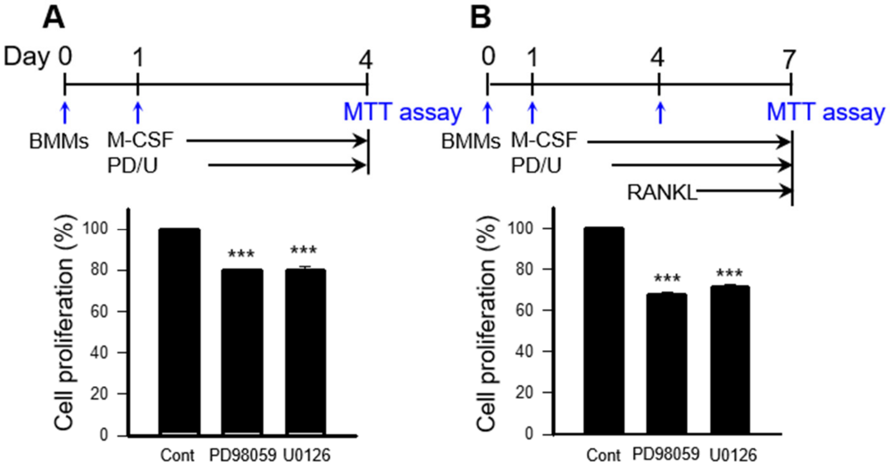

Figure S1: ERK inhibitors inhibited proliferation of BMMs. Cells (6×10⁴) were cultured with 30 ng/mL M-CSF for 3 days (Day 4) followed by with 30 ng/mL M-CSF and 50 ng/mL RANKL for 3 days (Day 7). Cells were treated with 5 µM PD98059 and 2 µM U0126 at the indicated time points, and proliferation was determined using thiazolyl blue tetrazolium bromide (MTT) reduction assay. Effect of ERK inhibitors PD98059 and U0125 on BMM proliferation during the first 3 days (n=4) (**A**) and for 7 days of culture (**B**). Data represent the mean ± SD (n = 4). ***p < 0.001 vs. control.

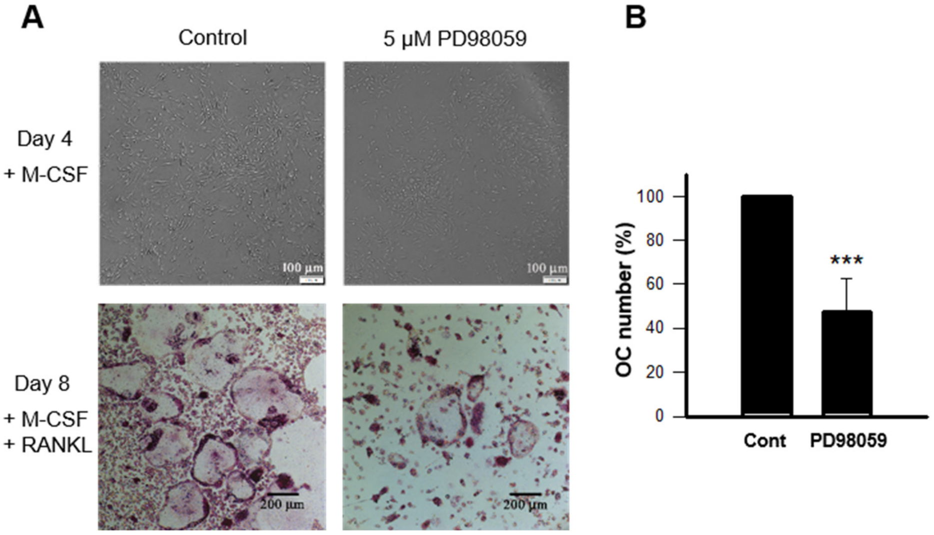

Figure S2: Inhibition of ERK decreased OC differentiation in BMMs. Cells (6×10⁴) were treated with 5 µM PD98059 in the presence of 30 ng/mL M-CSF for 3 days (Day 4) followed by treatment with 30 ng/mL M-CSF and 50 ng/mL RANKL for 4 days (Day 8). (**A**) Representative pictures of cell morphology on day 4, and TRAP-positive OCs on day 8. (**B**) Number of TRAP-positive multinucleated OCs on day 8 (n = 2 independent experiments, each performed in triplicate). ***p < 0.001 vs. control.
